# Investigation of associations between polycystic ovary syndrome and INSR gene polymorphisms rs2059806 and rs2252673

**DOI:** 10.1590/1806-9282.20241056

**Published:** 2025-03-17

**Authors:** Emre Taşkin, Semra Eroğlu

**Affiliations:** 1 Bandırma Onyedi Eylül University, Faculty of Medicine, Department of Medical Genetics – Balıkesir, Turkey.; 2 Samsun University, Samsun Practice Hospital, Faculty of Medicine, Department of Obstetrics and Gynecology – Samsun, Turkey.

**Keywords:** Polycystic ovary syndrome, Polymorphism, PCOS, Insulin receptor, Diabetes mellitus, Turkey

## Abstract

**OBJECTIVE:**

The etiology of polycystic ovary syndrome is still clearly unknown. Research results indicate that polycystic ovary syndrome may be a multifactorial disease whose inheritance pattern is potentially autosomal dominant. INSR gene polymorphisms are frequently seen among polycystic ovary syndrome patients who also have insulin resistance. The aim of this study was to investigate associations between INSR gene polymorphisms rs2059806 and rs2252673 with polycystic ovary syndrome.

**METHODS:**

A total of 48 polycystic ovary syndrome and 50 control subjects were recruited in this case-control study. A real-time polymerase chain reaction method (particularly the cycle threshold method) was used for polymorphism genotyping. Genotype and allele frequencies as well as the effects of the genotypes on having polycystic ovary syndrome were evaluated by appropriate statistical methods. Also, differences between genotypes in terms of clinical characteristics were tested.

**RESULTS:**

There was no difference in genotype and allele frequencies between the polycystic ovary syndrome and control groups when calculated under both additive and dominant models (p>0.05). The polycystic ovary syndrome group showed significantly higher mean testosterone levels (p<0.001) and significantly lower estradiol (p=0.006), follicle-stimulating hormone (p=0.021), and progesterone (p<0.001) levels compared to controls. The GG genotype (polymorphic) of the rs2252673 polymorphism in the polycystic ovary syndrome group showed significantly higher mean testosterone and progesterone levels compared to both GC and CC genotypes (p=0.004 and p=0.019, respectively).

**CONCLUSION:**

Being the first of its kind that investigates associations between polycystic ovary syndrome and INSR gene rs2059806 and rs2252673 polymorphisms in a population from Turkey, the present study detected no association.

## INTRODUCTION

According to the American Society for Reproductive Medicine and the European Society of Human Reproduction and Embryology (ESHRE/ASRM) consensus, polycystic ovary syndrome (PCOS) is described by three conditions, such as oligo-ovulation, hyperandrogenism, and ovaries with multiple cysts^1^. On the other hand, researchers have reported that anovulation is required for the diagnosis of PCOS^2^. Incidence rates vary worldwide, while globally they have been reported as 5-18% among women of reproductive age^3^. PCOS is also associated with other conditions such as insulin resistance, Type 2 diabetes mellitus (T2DM), dysglycemia, atherogenic dyslipidemia, obesity, and gestational diabetes^4^. Likewise, in a systematic review and meta-analysis involving nine studies, higher body mass index (BMI) was associated with PCOS^5^. Increasing evidence show the significant role of genetic and environmental factors in PCOS^6^. Moreover, family-based studies suggest that PCOS may be inherited by a dominant pattern^6^. In addition, first-degree PCOS patients tend to develop insulin resistance^7^. Genetic variations in the INSR gene have been shown to be associated with insulin resistance, which may lead to a predisposition to PCOS^8^. Also, it has been shown that mutations in the INSR gene cause hyperinsulinemia as well as insulin resistance^9^.

Due to the involvement of multiple organs and systems related to metabolism and the reproductive system, the genetic components of PCOS are considered to be heterogeneous; therefore, highly divergent results have been obtained from similar studies. Recently, it has been found that no studies investigating associations between rs2059806 and rs2252673 polymorphisms of the INSR gene and PCOS have been conducted on the Turkish population, and hence this study was conducted.

## METHODS

### Participant recruitment

All participants were diagnosed by Assoc. Prof. Dr. (MD) Semra Eroğlu. The inclusion criteria were having at least two of the following conditions that were identified in the Rotterdam criteria: oligo-ovulation, clinical or biochemical hyperandrogenism, and polycystic ovarian appearance in ultrasound images. The exclusion criteria were set as not being relatives and not having diabetes mellitus and other metabolic diseases. After an informed written consent was signed by the participants, 2 mL of venous blood was collected in EDTA-containing tubes. DNA was extracted using a blood DNA extraction kit (GF-1 Blood DNA Extraction Kit—Vivantis). Ethical approval was provided by the Ethics Committee of Karabuk University, in meeting number 3.

### DNA genotyping

The real-time polymerase chain reaction (PCR) cycle threshold (Ct) method with SYBR Green I dye was used for DNA genotyping (ABI 7500 Real-Time system). Custom primers were designed, which may be shared upon request from the contributing author. In the PCR step, two wells for one participant were used: in the first one, a wild-type primer and in the second one a polymorphic primer were mixed with the reaction mix. After the PCR step, in the melting analysis, genotype determination between the wild-type and polymorphic alleles was done by observing the T_m_ of the DNA. A volume of 25 μL of the total reaction mix was used. The evaluation of the real-time PCR results of the experiments was made using T_m_ graphics provided by the software.

### Statistical analysis

SPSS software was used for calculating statistics. The Hardy–Weinberg equilibrium could not be confirmed in the control group possibly due to a low sample size. The significance level of the statistics was determined as p<0.05. For calculating the differences between the PCOS and control groups in terms of clinical characteristics, Mann-Whitney U and Student's t-tests were used. Continuous variables were presented as mean±standard deviation (SD). The normal distributions of the continuous variables were tested with the Kolmogorov-Smirnov test. The gene counting method was used to determine the differences in genotype and allele frequencies. The Levene's test was used to test the homogeneity of variances. ANOVA test was used to evaluate differences in biochemical parameter levels between genotypes. A logistic regression analysis was performed to evaluate the effects of polymorphisms on PCOS.

## RESULTS

As shown in Table 1, testosterone levels were significantly higher in the PCOS group compared to the control group (p<0.001), whereas estradiol, follicle-stimulating hormone (FSH), and progesterone levels were significantly lower in the PCOS group compared to the control group (p=0.006, p=0.021, and p<0.001, respectively).

**Table 1:** Clinical characteristics of the polycystic ovary syndrome and control groups.

Characteristics	PCOS group (n=48)	Control group (n=50)	p
Age (years)	23.7±5.8	26.3±7.2	0.056
BMI (kg/m^2^)	24.5±6.0	25.1±4.6	0.0585
Insulin (pmol/L)	135±139	134±94.8	0.949
Testosterone level (nmol/L)	1.79±0.763	1.26±0.7	**<0.001**
Fasting glucose level (mmol/L)	5.13±0.055	5.19±0.85	0.683
Estradiol level (pmol/L)	60.1±36.1	92.3±70.1	**0.006**
FSH (IU/L)	6.94±2.08	9.99±8.77	**0.021**
LH (IU/L)	11.2±7.88	12.1±15.0	0.703
Progesterone (ng/mL)	1.18±2.38	4.64±4.02	**<0.001**
Prolactin (μg/L)	15.5±10.3	11.9±7.69	0.056
TSH (IU/L)	2.81±1.51	2.35±1.86	0.187

Only available data are included. Standard deviations are given after ± symbol. FSH: follicle-stimulating hormone. LH: luteinizing hormone. TSH: thyroid-stimulating hormone. PCOS: polycystic ovary syndrome. BMI: body mass index. Statistically significant values are denoted in bold.

The genotype and allele frequency distributions and logistic regression results are shown in Table 2. Under both additive and dominant models, there was no difference in genotype and allele frequencies between the PCOS and control groups (p>0.05). Based on either an adjusted or an unadjusted model, both polymorphic allele carriers and homozygous polymorphic genotypes did not have any effect on having PCOS.

**Table 2:** Regression results and genotype/allele frequency comparisons.

Polymorphism	Genotype	PCOS	Controls	p	Unadjusted regression	p	1Adjusted regression	p
	Additive model				OR (95%CI)		OR (95%CI)	
rS2059806, n (%)	AA	17 (35)	14 (28)	0.246	1		1	
	AG	14 (30)	10 (20)		1.153 (0.393-3.383)	0.8	1.635 (0.477-5.602)	0.43
	GG	17 (35)	26 (52)		0.538 (0.211-1.371)	0.19	0.472 (0.177-1.257)	0.13
Allele frequencies A/G		0.5/0.5	0.38/0.62	0.905				
rS2252673, n (%)	CC	24 (50)	28 (56)	0.830	1		1	
	CG	20 (42)	18 (36)		1.296 (0.561-2.998)	0.54	1.341 (0.550-3.269)	0.52
	GG	4 (8)	4 (8)		1.167 (0.263-5.173)	0.84	1.069 (0.226-5.044)	0.93
Allele frequencies C/G		0.71/0.29	0.74/0.26	0.62				
	**Dominant model**				**Unadjusted regression**		1 **Adjusted regression**	
					**OR (95%CI)**		**OR (95%CI)**	
rS2059806, n (%)	AA	17 (35)	14 (28)	0.43	1		1	
	AG+GG	31 (65)	36 (72)		0.709 (0.302-1.667)	0.43	0.695 (0.281-1.718)	0.43
rS2252673, n (%)	CC	24 (50)	28 (56)	0.552	1		1	
	CG+GG	24 (50)	22 (44)		1.273 (0.575-2.818)	0.55	1.288 (0.553-2.998)	0.56

1 Adjusted for age and BMI. BMI: body mass index; PCOS: polycystic ovary syndrome. OR: odds ratio. CI: confidence interval.

According to an intragroup clinical characteristic comparison between genotypes under an additive model in Table 3, in the PCOS group, the testosterone and progesterone levels of the GG genotype of the rs2252673 polymorphism were significantly higher compared to both AA and AG genotypes (p=0.004 and p=0.019, respectively). Again, under the additive model, in the PCOS group, the mean estradiol levels of the rs2059806 polymorphism were significantly different among genotypes (p=0.038).

**Table 3:** Intragroup clinical characteristic comparison of genotypes under an additive model.

		*rS2059806*			p	*rS2252673*			p
		AA	AG	GG		CC	CG	GG	
Controls	BMI	24±3.6	25±1.9	25.9±5.67	0.311	25.0±5.52	25.2±3.53	24.9±2.66	0.994
	Fasting glucose	96±23	89±4.3	94.2±12.8	0.502	94.2±17.9	93.1±12.9	91±4.24	0.919
	Insulin	29±23	15±7.3	21.4±12.0	0.097	21.7±19.1	22.0±6.89	27.4±22.7	0.801
	Testosterone	40±23	38±27	33.8±15.6	0.654	40,9±24.2	28.1±9.05	40.3±16.6	0.097
	Estradiol	114±100	101±61	76.9±51.2	0.26	88.3±83.7	96.3±52.6	102±50.0	0.898
	FSH	11±7.2	9.6±4.9	9.8±10.7	0.962	11.4±10.8	8.08±5.38	8.62±1.81	0.44
	LH	18±26	10±5.5	10.0±9.44	0.268	11.8±9.86	13.8±21.5	6.93±2.72	0.708
	Progesterone	56±3.9	3.6±2.5	4.3±4.49	0.339	4.90±4.48	4.28±3.67	4.46±2.46	0.879
	Prolactin	11.5 ± 7.8	16±12	10.7±5.26	0.194	11.3±9.05	12.3±5.73	15.4±4.81	0.607
	TSH	2.35±2.4	2.5±2.4	2.3±1.89	0.971	2.21±1.12	2.27±1.86	3.67±4.64	0.338
PCOS	BMI	26±7.4	23±4.1	24.5±5.9	0.493	24.9±5.31	24.6±6.92	21.4±5.94	0.566
	Fasting glucose	92±14	93±10	91.8±7.6	0.906	93.0±13.4	92.3±6.25	89.5±12.8	0.835
	Insulin	25±31	27±25	16.3±5.61	0.39	21.7±18.9	24.9±29.6	15.2±5.30	0.733
	Testosterone	53±17	52±19	50.1±29.1	0.929	48.7±17.6	48.6±19.8	85.5±32.9	**0.004**
	Estradiol	47±23	54±23	77.4±48.4	**0.038**	53.9±26.4	69.6±44.8	49.8±36.0	0.302
	FSH	7.1±1.7	6.5±1.9	7.2 ± 2.55	0.649	6.82±1.73	6.91±2.61	7.84±0.814	0.667
	LH	11.5±10	9±7	12.7±5.71	0.424	11.9±7.02	9.34±8.49	16.52±8.63	0.215
	Progesterone	0.6±0.4	1.1±1.5	1.83±3.70	0.315	0.88±1.19	0.92±1.28	4.32±7.28	**0.019**
	Prolactin	17±13	13±9.4	15.5±10.3	0.567	14.4±7.41	18.2±13.3	9.13±4.09	0.203
	TSH	2.5±1.25	3.3±1.9	2.68±1.39	0.297	2.64±1.81	3.11±1.18	2.35±0.777	0.494

Only available data are included. Standard deviations are given after the ± symbol. FSH: follicle-stimulating hormone. LH: Luteinizing hormone. TSH: thyroid-stimulating hormone. BMI: body mass index; PCOS: polycystic ovary syndrome. Statistically significant values are denoted in bold.

## DISCUSSION

In the literature, 12 polymorphisms in the INSR gene were investigated for associations with PCOS. Goodarzi et al.^10^ reported that among the genes of the insulin signaling pathway including *AKT2*, *INSR*, *IRS1*, *IRS2*, *GSK3B*, *PTP1B*, *PPP1R3*, and *SORBS1*, the most influential ones on PCOS were *INSR* and *ISR2*. The INSR gene was addressed to the chromosomal position 19p13.3-13.2 with 22 exons and consists of two subunits: an α-subunit which is encoded by 1-11 exons and a β-subunit which is encoded by 12-22 exons. The rs2252673 polymorphism is in the intron sequence, nonmodifying the amino acid sequence, although it may affect the transcription and/or splicing of the mRNA of the gene.

In the pathogenesis of PCOS, hyperandrogenemia is considered to play a role via hyperinsulinemia that is driven by hepatic sex hormone-binding globulin (SHBG) and increased free testosterone levels^11^. From the androgen gene perspective, in a review, the researchers pointed out that androgen receptor gene polymorphisms may be promising as biomarkers for PCOS^12^. We found that the mean testosterone level of the PCOS group was significantly higher than that of the control group (p<0.001). This was expected since it is a clinical outcome of PCOS. Whereas the estradiol, FSH, and progesterone levels of the PCOS group were significantly lower than those of the control group. This result was compatible with PCOS manifestations and a previous result that was reported from another ethnic population sample (p=0.006)^13^.

The rs2059806 polymorphism is a G-A alteration in the exon 8 of the *INSR* gene and was reported not to be associated with PCOS in two meta-analyses^14^. In our study, we did not find any association between the rs2059806 polymorphism and PCOS (p>0.05). Bagheri et al.^15^ also did not find any significant genotype and allele frequency difference of the rs2059806 polymorphism between PCOS patients and control participants. Their study has a limitation of low sample size, but they included Iranian Azeri Turkish women who are ethnically close to our participants. Ranjzad et al.^16^ did not also find any association between PCOS and the rs2059806 polymorphism in an Iranian cohort. A recent review in the literature related to PCOS and *INSR* polymorphisms was published by Feng et al.^14^ reporting that according to four study results with 524 PCOS patients and 442 controls, the rs2059806 polymorphism was not associated with PCOS, confirming previous studies and ours^14^.

In our study, we detected that in the PCOS group, the mean estradiol level of the GG genotype of the rs2059806 polymorphism under an additive model was significantly higher than that of the AA genotype according to post-hoc tests (p=0.037, data not shown). In the mentioned studies, there were no intragroup clinical characteristic comparisons between genotypes in the PCOS and control groups separately; therefore, we cannot compare our results directly. Estradiol is an important deterministic component of the PCOS clinical picture. Since we detected this difference only in the PCOS group, replication statistics from other studies are needed to confirm whether this significant difference in estradiol levels arises from polymorphic genotypes or any other factors that influence the clinical picture of the patients.

We did not observe any significant difference in genotype and allele frequencies between the PCOS and control groups in this study. Du et al.^17^ found in Han Chinese that, the GG genotype carriers of the rs2059806 polymorphism were significantly more prone to develop PCOS with a genotype frequency of 53.1% versus 43.2% (p<0.05) in controls, which is dissimilar to our regression test results (8% in both groups) (p>0.05)^17^. The G allele frequency in the same study was 72.5% for the PCOS group and 64.3% for the control group, indicating a significant difference (p<0.05), while our PCOS and control groups did not show any significant G allele frequency difference with percentages of 29 and 26%, respectively (p>0.05)^17^.

Du et al.^17^ did not encounter any significant difference in clinical characteristics between the genotypes of the rs2059806 polymorphism either in the PCOS or control group separately. However, after applying a recessive model (CC versus CG+GG) in the PCOS group, they determined that polymorphic allele carriers were associated only with higher total cholesterol and high-density lipoprotein-cholesterol (HDL-C) levels (p=0.048 and p=0.026, respectively). We could not detect any significant difference in either group in terms of both total cholesterol and HDL-C levels between genotypes (p>0.05, data not shown). However, in a study of PCOS phenotypes, it was reported that among a phenotype sub-group of PCOS patients, the estimated average glucose was predicted by higher levels of HDL-C^18^. Under the additive model in our PCOS group, we found that the testosterone and progesterone levels of the GG genotype of the rs2252673 polymorphism were significantly higher than those of both CC and CG genotypes, a result that shows the influence of the mentioned polymorphisms on hormonal imbalance among PCOS patients (p=0.004 and p=0.019, respectively).

According to 1000 Genomes^19^, the C allele frequency of the rs2252673 polymorphism varies from 0.159 to 0.726 in different countries worldwide (e.g., in Koreans, the minor one is C allele, while in Caucasians, the major allele is C), while in our study, we found that it was 0.29 for PCOS and 0.26 for controls, which is similar to an European population that varies from 0.159 to 0.247^10,20^. Considering that the ethnicity of this study is close to that of the European population, this result is expected since minor allele frequencies go gradually higher toward the Eastern countries^19^. On the other hand, contrary to our results, in a Korean population, Lee et al.^20^ found that carriers of the G allele (which is the major allele in Koreans) are more prone to develop PCOS.

Similar to other studies, we also intended to see whether BMI levels are significantly different between genotypes since PCOS patients may have a tendency to become obese as a result of insulin resistance and T2DM^17,21^. Although all the study participants were lean, we checked for any associations of genotype and allele frequencies with BMI under both dominant and additive models. However, we could not find any significant BMI difference among both the gene rs2059806 and rs2252673 polymorphisms (p>0.05). Also, Goodarzi et al.^10^ did not find any association between BMI and the rs2252673 polymorphism in PCOS patients. On the other hand, Soares-Jr. et al.^22^ investigated the influence of PCOS phenotypes on metabolic syndrome and found that individuals with phenotype A (among A, B, C, and D) were at a higher risk of T2DM. Although the major limitation of this study is a low sample size, to our knowledge, this is the first study investigating associations between PCOS and INSR gene rs2059806 and rs2252673 polymorphisms in a population from Turkey. Another limitation of our study is the lack of grouping participants with sub-phenotypes according to the Rotterdam PCOS diagnostic criteria.

## CONCLUSION

INSR gene rs2059806 and rs2252673 polymorphisms are not associated with PCOS in the studied population sample.

This study was approved by the Ethics Committee of Karabük University—Faculty of Medicine, on October 26, 2016, in meeting number 3.
